# Addictions in the elderly: clinical characteristics, challenges, and management strategies

**DOI:** 10.1192/j.eurpsy.2026.11560

**Published:** 2026-07-31

**Authors:** F. Zaouali, I. Anes

**Affiliations:** 1 Emergency department and mobile emergency and resuscitation service, Tahar Sfar University Hospital, Mahdia; 2 Outpatient psychiatric consultation, Hadj Ali Soua Regional Hospital, Ksar Hellal, Monastir, Tunisia

## Abstract

**Introduction:**

Addiction among the elderly is an emerging public health concern often overlooked due to diagnostic complexities and stigma. With increasing life expectancy, substance use disorders and behavioral addictions in older adults present unique clinical challenges, including comorbid medical conditions, polypharmacy, and atypical presentations.

**Objectives:**

This study aims to explore the prevalence, clinical features, and management approaches for addictions in patients aged 65 and above.

**Methods:**

A retrospective study was conducted on patients aged 65 years and older diagnosed with addiction disorders over a six-year period (2018-2023) at the outpatient consultation at hadj Ali Soua regional hospital of Ksar Hellal, Monastir, Tunisia. Data collected included sociodemographic variables, types of addiction (alcohol, prescription medications, illicit substances, behavioral addictions), comorbid psychiatric and medical conditions, treatment modalities, and clinical outcomes. Descriptive statistics and qualitative analysis were performed.

**Results:**

Among 48 elderly patients identified with addiction, alcohol use disorder was predominant (58%), followed by prescription drug misuse (25%) and behavioral addictions such as gambling (17%). Common comorbidities included depression (46%), cognitive impairment (29%), and chronic pain (38%). Treatment involved integrated pharmacological and psychosocial interventions tailored to the elderly, including motivational interviewing and cognitive-behavioral therapy. Clinical outcomes indicated moderate improvement in 65% of cases, with challenges including medication adherence and social isolation.

**Conclusions:**

Addictions in older adults require increased clinical awareness and specialized management strategies considering their unique physiological and psychosocial context. Early detection and multidisciplinary approaches can improve outcomes and quality of life. Further research is needed to develop age-appropriate screening tools and treatment protocols.

**Disclosure of Interest:**

None Declared

